# Identification and prioritization of macrolideresistance genes with hypothetical annotation inStreptococcus pneumoniae

**DOI:** 10.6026/97320630014488

**Published:** 2018-12-09

**Authors:** Blue Goad, Laura K Harris

**Affiliations:** 1 Davenport University, 200 S. Grand Ave, Lansing, MI, 48933, USA

**Keywords:** resistance genes, hypothetical genes, *Streptococcus pneumoniae*

## Abstract

Macrolide resistant Streptococcus pneumoniae infections have limited treatment options. While some resistance mechanisms are well established, ample understanding is limited by incomplete genome annotation (hypothetical genes). Some hypothetical genes encode a domain of unknown function (DUF), a conserved protein domain with uncharacterized function. Here, we identify and confirm macrolide resistance genes. We further explore DUFs from macrolide resistance hypothetical genes to prioritize them for experimental characterization. We found gene similarities between two macrolide resistance gene signatures from untreated and either erythromycin- or spiramycin-treated resistant Streptococcus pneumoniae. We confirmed the association of these gene sets with macrolide resistance through comparison to gene signatures from (i) second erythromycin resistant Streptococcus pneumoniae strain, and (ii) erythromycin-treated sensitive Streptococcus pneumoniae strain, both from non-overlapping datasets. Examination into which cellular processes these macrolide resistance genes belong found connections to known resistance mechanisms such as increased amino acid biosynthesis and efflux genes, and decreased ribonucleotide biosynthesis genes, highlighting the predictive ability of the method used. 22 genes had hypothetical annotation with 10 DUFs associated with macrolide resistance. DUF characterization could uncover novel co-therapies that restore macrolide efficacy across multiple macrolide resistant species. Application of the methods to other antibiotic resistances could revolutionize treatment of resistant infections

## Background

Streptococcus pneumoniae (S. pneumoniae) infections cause approximately 1.2 million life-threatening illnesses resulting in 7,000 deaths annually including bacterial upper respiratory infections and pneumonia and meningitis, and bloodstream, ear, and sinus infections 1,2. Macrolide antibiotics (i.e. erythromycin and spiramycin) are first line treatments for S. pneumoniae infections 3 that function by binding reversibly to the 50S bacterial ribosomal subunit, preventing protein synthesis (translation). Unfortunately, full macrolide resistance in S. pneumoniae, defined clinically by a minimum inhibitory concentration (MIC) greater than 256 mg/mL, is increasingly common 3 with approximately 30% of severe S. pneumoniae cases being fully resistant to one or more clinically relevant antibiotics 1. This makes antibioticresistant S. pneumoniae a serious public health threat 1

Several macrolide resistance mechanisms have been reported 2. Known macrolide resistance mechanisms include (1) ribosomal modification, for example via the ermB gene which is responsible for 50S subunit methylation, (2) efflux proteins encoded by major facilitator super-family ormef (E/A), mel, and msrD genes, and (3) ribosomal nucleic acid mutations, which can be either a simple point mutation causing a single amino acid change, such as lysine- 63-glutamaine in the ribosome rplD gene, a deletion of three amino acids (Met-82, Lys-83, and Glu-84) from rplV, or a variety of additional L4 and L22 ribosomal mutations 2-4. Dual macrolide resistance genotypes our understanding of direct resistance mechanisms further, some macrolide resistant isolates use one of these mechanisms while others use multiple mechanisms with no clear connection to level of resistance 2. Therefore, despite a good understanding of various direct macrolide resistance mechanisms, co-therapies to overcome macrolide resistance have yet to be established.

Mutant library studies have revealed large numbers of genes that both directly, as discussed prior, and indirectly influence drug resistance with many of these genes not clearly involved in known drug-resistant mechanisms 5. Indirect mechanisms associated with resistance can be metabolic, such as decreased Kreb's (i.e. TCA) cycle in vancomycin intermediate resistant Staphylococcus aureus 6. One way to uncover genes functioning indirectly with antibiotic resistance mechanisms is to examine gene expression and identify genes with markedly different expression (differentially expressed) between two conditions for further examination (i.e. hypothesis generation). This approach has been used to successfully predict antibiotic resistance in some bacterial pathogens such as Escherichia coli 5, but its application to other organisms like S. pneumoniae has been slow. Identifying differentially expressed genes associated with antibiotic resistance is a first step in fully elucidating the interaction between direct and indirect drug-resistance mechanisms.

Incomplete genome annotation substantially limits gene expression analysis 7, and is common for bacterial genomes 4, 8 with up to 50% of some bacterial genomes lacking annotation 9. A hypothetical gene is defined by its sequence alone, having little to no experimental evidence of its function, and lacking homology to genes with known function 4, 9. There are two types of hypothetical proteins: (i) uncharacterized protein families that lack domain information and are not usually conserved across phylogenetic lineages, and (ii) domains of unknown function (DUFs), functionally uncharacterized protein sections that have been shown to play essential roles in bacterial processes 9, 10. Over 20% of protein domains have DUFs annotations with around 2,700 DUFs found in bacteria and more than 800 DUFs shared between the domains of life 10. Identifying hypothetical genes associated with antibiotic resistance and prioritizing them for experimental characterization, such as structural determination 7, could lead to the development of life-saving co-therapies to preclude or overcome antibiotic resistance.

**Figure 1:**
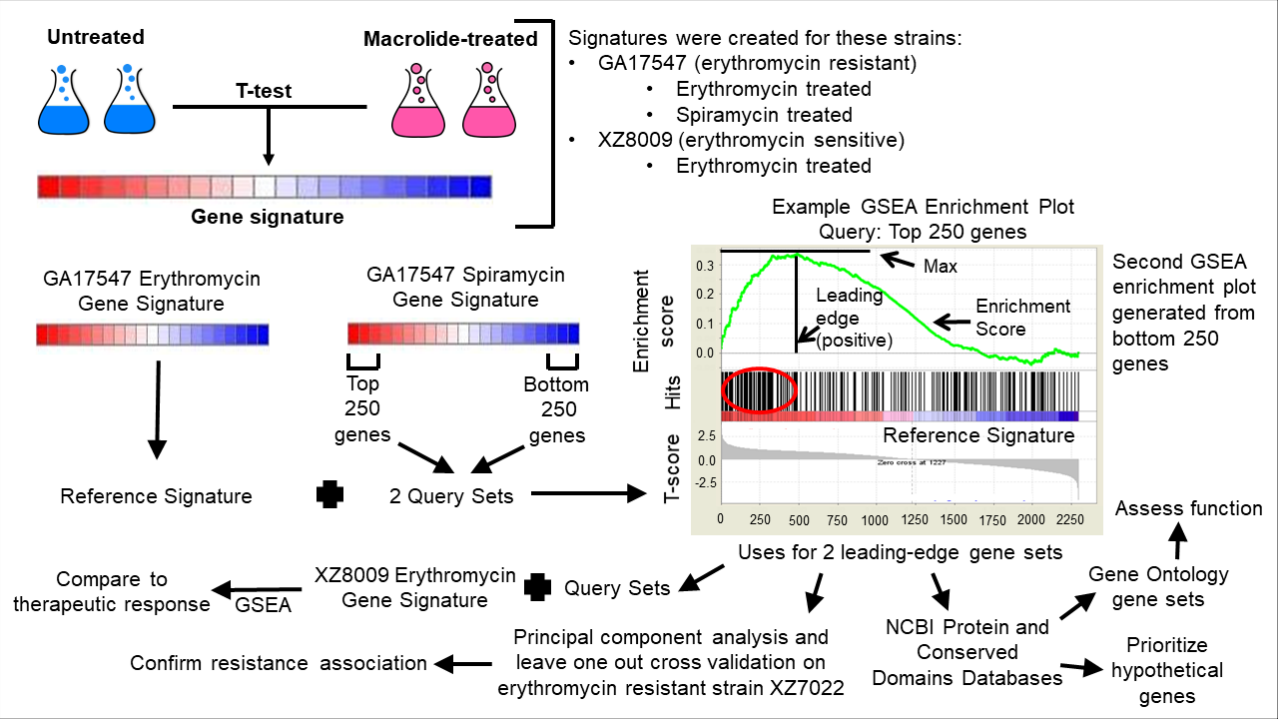
Schematic representation of study approach. Signatures (ranked list of genes from high to low expression) are created by ranking genes in expression dataset by T-score calculated by comparing untreated and macrolide-treated (either erythromycin or spiramycin) samples collected during mid-log phase of growth. To identify macrolide resistance genes, the erythromycin signature from erythromycin resistant S. pneumoniae strain GA17547 was used as reference and the top and bottom 250 genes from the GA17547 spiramycin signature were used as query gene sets (unranked list of genes with biological relevance) for Gene Set Enrichment Analysis (GSEA) comparison. Gene matches (hits) between the reference signature and query gene set being compared that contribute most to are grouped together as a leading-edge gene set. Leading-edge gene sets were then used (i) as query gene sets for GSEA comparison against a erythromycin gene signature from erythromycin sensitive strain XZ8009 (reference), (ii) to select genes for principal component analysis and leave one out cross validation on erythromycin resistant strain XZ7022, and (iii) for functional analysis by collecting gene symbols, descriptions, and protein domain information from National Center for Biotechnology Information (NCBI) databases then using Gene Ontology to assess lists for overrepresentation to known biological processes gene sets and prioritizing genes without symbols (hypothetical genes) by domain of unknown function detection.

In this paper, we identify and validate genes associated with macrolide resistance by comparing therapeutic response gene expression signatures (list of genes ranked from high to low differential expression between untreated and macrolide treated samples) in S. pneumonia (Figure 1). We noticed these genes were associated with known mechanisms of macrolide resistance, such as efflux, showing our approach�s ability to identify potential cotherapies to overcome macrolide resistance. However, as anticipated, 22 out of 160 (13.75%) macrolide resistance genes identified had hypothetical annotation. To address this, we examined hypothetical genes for DUFs and propose prioritized gene targets related to macrolide resistance for immediate experimental characterization. Through we introduce this approach while exploring erythromycin resistant S. pneumoniae, recommendations provided by applying our approach to other antibiotic resistant bacterial infections can reduce development costs and time to availability for potential new co-therapy targets, substantially renovating the way antibiotic resistant infections are treated clinically.

## Methodology

### Gene expression datasets

Macrolide resistant S. pneumoniae samples used in this study came from the Gene Expression Omnibus (GEO) accession GSE54176, which included samples of untreated (n=6) and macrolide treated (1.2mg/L erythromycin n=6, 0.1mg/L spiramycin n=3) macrolide resistant (GA17547, n=2/condition, 6 samples total, and XZ7022, 2 for erythromycin and vehicle, 1 for spiramycin, 5 samples total) and sensitive (XZ8009, n=2 for erythromycin and vehicle, 4 samples total) strains 3, 11, 12. Gene expression data was used as provided by GEO without alteration.

Identification and validation of macrolide resistance genes Using these expression data, we created erythromycin and spiramycin gene signatures (ranked by T-score) for macrolide resistant GA17547 strain (Figure 1). We used the erythromycin signature as reference and 250 most over- or under-expressed spiramycin genes as query gene sets for Gene Set Enrichment Analysis (GSEA), which calculates a running summation (enrichment score) based on the T-score of matches (hits) between the reference signature and query gene sets 13. From this, we can (i) estimate how similar these signatures are (significance) by calculating a normalized enrichment score (NES) and p-value from 1000 gene permutations, and (ii) identify genes that contribute to achieving maximum enrichment score (i.e. leading-edge genes) associated with macrolide resistance. Leading-edge (93 over- and 67 under-expressed, Table 1 and Table 2, respectively) genes are used for further analysis.

To confirm that identified leading-edge genes are related to resistance, we (i) used leading-edge genes as query gene sets for GSEA with a T-ranked erythromycin response signature from a macrolide sensitive strain (XZ8009) as reference, and (ii) utilized Principal Component Analysis (PCA) and Leave One Out Cross Validation (LOOCV) to examine expression of leading-edge genes in another macrolide resistant strain, XZ7022 (Figure 1). PCA is an unsupervised dimensionality reduction machine-learning technique that visualizes high-dimensional datasets (in our case 67 and 93 dimensions) in 2D space. PCA considers all samples of high dimensional and converts them into principal components, a smaller number of uncorrelated variables. When principal components are plotted in 2D space, variation between samples is observed as separation along principal components. Alternatively, LOOCV will set aside each sample individually (i.e. test set) and calculates a multiple linear regression equation from the remaining samples (i.e. training set). The resulting equation is used to predict the treatment condition of the test set sample. This process is repeated until all samples are left out and accuracy determined from the results.

### Functional association and hypothetical gene identification from macrolide resistance genes

To identify cellular processes associated with our leading-edge genes, we utilized the Panther search feature 14 at the Gene Ontology (GO) knowledgebase 15, 16, accessed October 17, 2018 (Figure 1). Panther calculates a p-value using the Fisher's Exact Test for each user-inputted gene set compared to established gene sets in the GO knowledgebase. For this comparison, we converted each leading-edge gene's locus tag provided by GEO to a gene symbol. To do this, we queried the Protein database from the National Center for Biotechnology Information (NCBI) for each locus tag and collected gene symbols from the connected Conserved Domains Database 17. If a locus tag did not have domains in the Conserved Domains Database, we verified its hypothetical status by examining homologs identified via Basic Local Alignment Search Tool Protein (BLASTP) 18. Symbols for all leading-edge genes without exception were included in GO analysis.

## Results and Discussion

### Similarities between erythromycin and spiramycin signatures reveal genes associated with macrolide resistance

**Figure 2:**
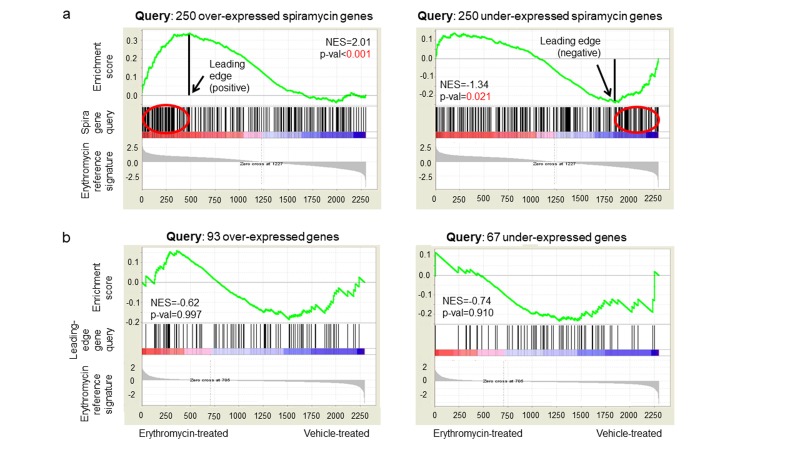
Similarities detected between erythromycin (reference) and spiramycin (spira, query gene sets) signatures from an erythromycin resistant S. pneumoniae strain, revealing leading-edge genes. (a) Differential expression of leading-edge genes (query gene sets) was not a response erythromycin treatment as seen by comparison to a erythromycin signature from a macrolide sensitive S. pneumoniae strain (reference). (b) These findings suggest identified leading-edge genes are associated with macrolide resistance rather than response to macrolide treatment.

To identify genes associated with macrolide resistance, we compared erythromycin and spiramycin gene expression signatures with the idea that genes with similar differential expression when erythromycin resistant S. pneumoniaeis treated with different macrolides are associated with macrolide resistance. We observed a statistically significant similarity between erythromycin and spiramycin signatures (p<0.22, Figure 2a). Of the 250 most over- and under-expressed genes from the spiramycin signature used as query gene sets for comparison to the erythromycin signature, 93 over- and 67 under-expressed genes were identified as contributing most to achieving maximum enrichment score (i.e. leading-edge, Table 1 and Table 2, respectively). We then used each leading-edge gene set as query for GSEA against an erythromycin response signature from a macrolide sensitive S. pneumoniae strain with the hypothesis that these genes would not be differentially expressed in response to macrolide treatment. We observed a relatively random distribution of leading-edge genes across the macrolide response signature (p> 0.900, Figure 2b), supporting their role in resistance rather than their expression changing as a response to treatment. These genes may contribute to macrolide resistance and become valuable reverse macrolide resistance therapeutic targets.

**Figure 3:**
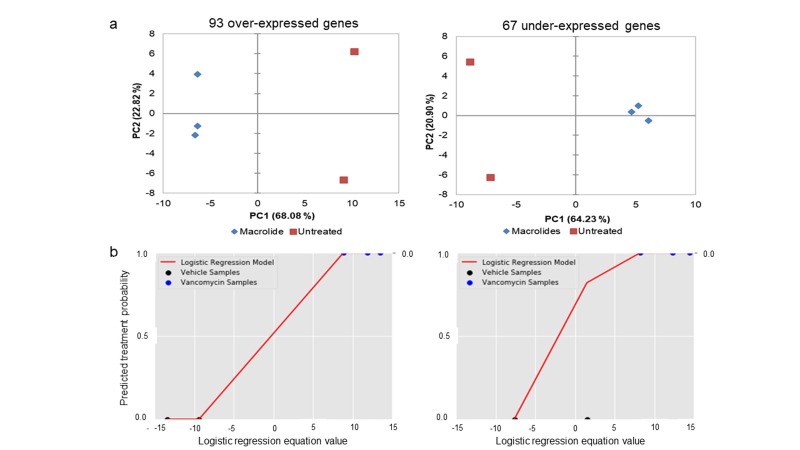
Principal component analysis separates erythromycin resistant samples based on treatment using gene expression values of 93 over- (left) and 67 under-expressed (right) leading-edge genes when plotted along two principal component (PC) that account for the most variation (in parentheses) across samples (a). Leave one out cross validation was used to quantify this separation for both leading-edge gene sets (b). One sample is removed (i.e. test set) and a multivariable logistic regression equation is computed for remaining samples (i.e. training set). The equation is then applied to the test set and based on the value (positive or negative) the program predicts treatment of the test set. Predicted equation values for each test set are plotted (colored dots along x-axes). The average coefficients for each gene across all training set equations is used to create a master equation used to predict treatment of all samples individually and calculate each prediction's probability of accuracy (red line).

To confirm that the macrolide resistance genes (leading-edge) we identified are related to resistance, we used PCA to see if expression of these genes in a non-overlapping dataset from a related erythromycin resistant S. pneumoniae strain could separate samples based on treatment (marcrolide or untreated). Both overand under-expressed leading-edge gene sets were able to separate macrolide treated from untreated samples, regardless of which macrolide (erythromycin or spiramycin) was used for treatment (Figure 3a). To quantify this separation ability, we used LOOCV on the same erythromycin resistant S. pneumoniae strain dataset. Multiple linear regression equations derived from these data were successfully able to predict treatment of left out samples with 100% and 80% accuracy for over- and under-expressed leading-edge genes, respectively (Figure 3b). While the sample size used in this study is small and we acknowledge that inclusion of more samples would make findings more robust and prevent over fitting, these results support the conclusion that our leading-edge genes are related to macrolide resistance.

### Macrolide resistance genes involved in increased amino acid biosynthesis and decreased ribonucleotide synthesis

To identify which cellular processes our macrolide resistant genes (i.e. leading-edge) correspond to most, we compared leading-edge gene lists to gene lists of known biological processes from GO to assess for over-representation via Fisher's Exact Test 14-16 [14-16]. GO identified 7 of 93 over-expressed leading-edge genes were related to amino acid biosynthesis: dapA, asd, alr, proC, proB, ilvE, and glyA (p=0.024). We noted genes clustered into several processes not identified as over-represented by GO such as 20 transporter genes 3 (7 ABC transporters with 2 related to antibiotic resistance, 4 efflux genes with 2 belonging to the major facilitator super family which is responsible for macrolide efflux 1 [19], and 9 miscellaneous transporter genes) and 8 translation-related genes (including 4 ribosomal subunits). Further, we observed 12 of 67 under-expressed leading-edge genes were associated with organic substance metabolism: ccpA, arc, dltC, sdaA, rnhB, uvrC, lacA, pgi, pyrC, purC, hpt, and upp (p=0.005), with 5 of those genes specializing in ribonucleotide synthesis: pgi, pyrC, purC, hpt, and upp (p=0.014). This shows the capability of our approach to detect known mechanisms of resistance that may or may not be identified by GO 19.

### Cellular process detection is limited by incomplete genome annotation

Incomplete genome annotation is a wide-spread challenge to examining cellular processes in bacteria 20. Unfortunately, this study was not immune to this major limitation as we observed 6 of 93 (6.5%) over-expressed and 10 of 67 (14.9%) under-expressed leading-edge genes had hypothetical annotation and confirmed their annotation via BLASTP. Since true hypothetical proteins require experimental investigation, we explored genes with hypothetical annotation further to provide useful recommendations for experimental endeavors. Following such guidance would maximize the potential to identify targets for new therapeutics that preclude and overcome macrolide resistance while minimizing experimental exploration costs.

To this end, we noted 3 over-expressed and 5 under-expressed hypothetical genes encoding DUFs (over-expressed: DUF979, DUF969, DUF389; under-expressed: DUF1447, DUF3042, DUF4649, DUF3884, DUF4231). Since DUFs span across domains of life 20, we recommend prioritizing these DUFs for experimental characterization since they may be involved in resistances in other life-threatening infections. Several hypothetical genes related to macrolide resistance did not have domain information yet (overexpressed: SPAR46_0613, SPAR46_1604, SPAR46_1486; underexpressed: SPAR46_0494, SPAR46_1297, SPAR46_0497, SPAR46_1346, SPAR46_1159). We recommend characterizing these over other hypothetical genes found in S. pneumoniae genomes because of their resistance connection.

## Conclusion

We identified and confirmed macrolide resistance genes in S. pneumoniae that are involved in increased amino acid biosynthesis and decreased ribonucleotide synthesis. Reversing activity for these cellular processes may overcome macrolide resistance. We noted that incomplete genome annotation (i.e. hypothetical genes) is a limitation to our analysis and further explored hypothetical genes related to macrolide resistance to recommend DUFs that are a priority for experimental characterization such as structural determination via nuclear magnetic resonance or X-ray crystallography. Characterization of DUFs identified here has the potential to uncover novel co-therapies that reverse macrolide resistance 7, restoring efficacy, not only for S. pneumoniae patients, but across multiple macrolide resistant species, saving thousands of lives annually.

Our gene signature comparison approach to identify DUFs associated with antibiotic resistance is a novel way to prioritize hypothetical genes for experimental characterization. Application of our approach across resistant bacterial infections would be valuable in reducing experimental time and financial costs for identifying new therapeutic targets. However, a major hindrance in these efforts is the availability of datasets run on the same platform. Variations in platforms used in gene expression studies require the use of gene symbols, reducing signature similarities and resulting in detection loss. Regardless, gene expression datasets for antibiotic resistant bacteria using the same platform are publicly available with more being deposited regularly. Results from further analysis could hold far-reaching advancements in treated antibiotic resistant infections globally.

## Figures and Tables

**Table 1:** Over-expressed leading-edge genes

Functional Categories1	Locus tag	Gene symbol	Gene description	Size (amino acids)
Alpha-amino acid biosynthetic process genes -7.5%	SPAR46_1029	dapA	dihydrodipicolinate synthase	311
	SPAR46_1028	asd	aspartate-semialdehyde dehydrogenase	358
	SPAR46_1697	alr	alanine racemase	367
	SPAR46_0936	proC	pyrroline-5-carboxylate reductase	265
	SPAR46_0934	proB	glutamate 5-kinase	369
	SPAR46_0842	ilvE	branched-chain amino acid aminotransferase	340
	SPAR46_1037	glyA	serine hydroxymethyltransferase family protein	418
ABC transporter genes (7.5%)	SPAR46_2172	pstC	phosphate ABC transporter, permease protein	287
	SPAR46_0811	glnQ	ABC transporter family protein	240
	SPAR46_1501	glnQ	ABC transporter family protein	209
	SPAR46_1502	hisM	ABC transporter protein	213
	SPAR46_2048	yadH	ABC-2 type transporter family protein	195
	SPAR46_1653	macA	ABC transporter family protein � macrolide resistance	171
	SPAR46_1352	mdlB	ABC transporter transmembrane region family protein � multi-drug resistance	582
Efflux genes (4.3%)	SPAR46_0355	rlmL	methyltransferase small domain protein	385
	SPAR46_0253	rlmL	acetyltransferase family	231
	SPAR46_0980	mdtG	major facilitator superfamily	399
	SPAR46_1610	MFS1	major facilitator superfamily	383
Miscellaneous transporter genes -9.6%	SPAR46_1772	secY2	accessory Sec system translocase SecY2	405
	SPAR46_0224	secY	preprotein translocase, SecY subunit	436
	SPAR46_1562	fieF	cation diffusion facilitator transporter family	367
	SPAR46_0733	livM	branched-chain amino acid transport system / permease component family	318
	SPAR46_0835	yufQ	branched-chain amino acid transport system / permease component family	272
	SPAR46_0162	metP	binding--dependent transport system inner membrane component family	230
	SPAR46_1922	dppC	binding--dependent transport system inner membrane component family	308
	SPAR46_0311	manZ	PTS system mannose/fructose/sorbose IID component family	272
	SPAR46_1888	pnuC	nicotinamide mononucleotide transporter	268
Exported product genes (3.3%)	SPAR46_0331	mpa1	polysaccharide export, MPA1 family	230
	SPAR46_0349	oppA	bacterial extracellular solute-binding subunit, 5 middle family	660
	SPAR46_1389	hisJ	bacterial extracellular solute-binding subunit, 3 family	271
Translation process genes (8.6%)	SPAR46_0205	rplD	50S ribosomal protein L4	207
	SPAR46_0207	rplB	ribosomal protein L2	277
	SPAR46_0217	rpsN	ribosomal S14p/S29e family protein	89
	SPAR46_0218	rpsH	ribosomal S8 family protein	132
	SPAR46_0532	infB	translation initiation factor IF-2	624
	SPAR46_1609	truA	tRNA pseudouridine synthase A	249
	SPAR46_1740	fmt	methionyl-tRNA formyltransferase	311
	SPAR46_1799	prmA	ribosomal protein L11 methyltransferase	316
Genes with duplicate descriptions (8.6%)	SPAR46_2127	matE	matE family protein	260
	SPAR46_2128	matE	matE family protein	166
	SPAR46_1311	crcB	crcB-like family protein	42
	SPAR46_1312	crcB	crcB-like family protein	124
	SPAR46_1350	ssnA	amidohydrolase family protein	419
	SPAR46_2182	ykuR	amidohydrolase family protein	376
	SPAR46_1183	yigB	HAD hydrolase, IA, variant 1 family protein	237
	SPAR46_2126	ysbA	HAD hydrolase, IA, variant 1 family protein	206
Miscellaneous leading-edge genes (35.5%)	SPAR46_0591	rnjA	RNA-metabolizing metallo-beta-lactamase family protein	553
	SPAR46_1277	topA	DNA topoisomerase I	648
	SPAR46_2293	ctsR	transcriptional regulator CtsR	152
	SPAR46_0151	mutR	transcriptional activator, Rgg/GadR/MutR family	287
	SPAR46_1018	His	histidine triad domain protein	189
	SPAR46_2342	yesM	histidine kinase-, DNA gyrase B-, and HSP90-like ATPase family protein	441
	SPAR46_1254	zwf	glucose-6-phosphate dehydrogenase	495
	SPAR46_1065	amyA	alpha amylase, catalytic domain protein	579
	SPAR46_0033	cynT	carbonic anhydrase family protein	165
	SPAR46_1608	thiD	phosphomethylpyrimidine kinase family protein	87
	SPAR46_0592	frmB	phospholipase/carboxylesterase family protein	259
	SPAR46_0524	cotS	phosphotransferase enzyme family protein	243
	SPAR46_1778	rfaJ	glycosyl transferase 8 family protein	315
	SPAR46_1173	aceF	2-oxoacid dehydrogenases acyltransferase family protein	347
	SPAR46_0408	fabH	3-oxoacyl-[acyl-carrier-] synthase III family protein	324
	SPAR46_1564	pcnB	poly A polymerase head domain protein	394
	SPAR46_0378	bre	phage integrase family protein	101
	SPAR46_0385	cvpA	colicin V production family protein	182
	SPAR46_1102	cgtA	obg family GTPase CgtA	436
	SPAR46_0982	rnr	ribonuclease R	784
	SPAR46_1741	priA	primosomal protein N	798
	SPAR46_2124	marR	marR family protein	141
	SPAR46_0264	ssuD	luciferase-like monooxygenase family protein	349
	SPAR46_0285	ydiL	CAAX amino terminal protease self- immunity family	235
	SPAR46_0511	pncU	bacteriocin-type signal sequence domain protein	82
	SPAR46_1993	cinA	competence or damage-inducible CinA	418
	SPAR46_2180	glpG	rhomboid family protein	225
	SPAR46_2181	fau1	5-formyltetrahydrofolate cyclo-ligase	179
	SPAR46_2335	yhgE	yhgE or Pip C-terminal domain protein	104
	SPAR46_0953	istB	istB-like ATP binding family protein	322
	SPAR46_0983	smpB	ssrA-binding protein	155
	SPAR46_1275	cutC	cutC family protein	210
	SPAR46_1300	lemA	lemA family protein	186
Genes needing improved description (-8.6%)	SPAR46_0361	COG5263	cell wall binding repeat family protein	332
	SPAR46_1800	NUDIX	NUDIX domain protein	142
	SPAR46_0330	ywqE	PHP domain protein	243
	SPAR46_0034	prsW	putative membrane protein	219
	SPAR46_2130	gcs2	hypothetical protein	425
	SPAR46_2110	comGF	hypothetical protein	153
	SPAR46_0951	cvfB	hypothetical protein	284
	SPAR46_1607	polY	hypothetical protein	81
Hypothetical genes (6.5%)	SPAR46_0844	HP	hypothetical protein - DUF969	104
	SPAR46_0846	HP	hypothetical protein - DUF979	307
	SPAR46_1280	HP	hypothetical protein - DUF389	145
	SPAR46_1604	HP	hypothetical protein - no domain information	99
	SPAR46_1486	HP	hypothetical protein - no domain information	40
	SPAR46_0613	HP	hypothetical protein - no domain information	86

**Table 2:** Under-expressed leading-edge genes

Functional Categories1	Locus tag	Gene symbol	Gene description	Size (amino acids)
Organic substance metabolism genes (17.9%)	SPAR46_2156	pgi	phosphoglucose isomerase family protein	426
	SPAR46_1178	pyrC	amidohydrolase family protein	323
	SPAR46_0059	purC	phosphoribosylaminoimidazolesuccinocarboxamide synthase	235
	SPAR46_2363	hpt	hypoxanthine phosphoribosyltransferase	180
	SPAR46_0728	upp	uracil phosphoribosyltransferase	209
	SPAR46_2045	ccpA	catabolite control protein A	336
	SPAR46_2243	arcC	carbamate kinase	315
	SPAR46_2269	dltC	D-alanine--poly(phosphoribitol) ligase, subunit 2	79
	SPAR46_0121	sdaA	serine dehydratase alpha chain family protein	132
	SPAR46_1166	rnhB	ribonuclease HII family protein	260
	SPAR46_1894	uvrC	enterocin A Immunity family protein	98
	SPAR46_1201	lacA	galactose-6-phosphate isomerase, LacA subunit	141
ABC transporter genes (6.0%)	SPAR46_0910	lolD	ABC transporter family protein	212
	SPAR46_0499	ccmA	ABC transporter family protein	243
	SPAR46_1716	ccmA	ABC transporter family protein	231
	SPAR46_1588	AAA	ABC transporter family protein	376
Duplicate genes (6.0%)	SPAR46_0128	pspA	cell wall binding repeat family protein	607
	SPAR46_0365	pspA	cell wall binding repeat family protein	40
	SPAR46_0603	rhoD	rhodanese-like domain protein	50
	SPAR46_0084	rhoD	rhodanese-like domain protein	98
Miscellaneous leading-edge genes (40.3%)	SPAR46_2241	arcA	arginine deiminase	409
	SPAR46_1930	ugpB	bacterial extracellular solute-binding family protein	419
	SPAR46_1929	ugpA	binding-dependent transport system inner membrane component family protein	288
	SPAR46_1333	lplB	binding-dependent transport system inner membrane component family protein	310
	SPAR46_0095	ompR	transcriptional regulatory family protein	232
	SPAR46_0607	HIT	HIT domain protein	153
	SPAR46_0608	ldcB	D-alanyl-D-alanine carboxypeptidase family protein	238
	SPAR46_0674	ftsQ	POTRA domain, FtsQ-type family protein	406
	SPAR46_0729	clp	clp protease family protein	196
	SPAR46_0999	acm	glycosyl hydrolases 25 family protein	250
	SPAR46_1135	degV	EDD, DegV family domain protein	279
	SPAR46_1136	himA	bacterial DNA-binding family protein	91
	SPAR46_1218	entA	enterocinA Immunity family protein	99
	SPAR46_1337	insE	transposase family protein	178
	SPAR46_1498	manB	phosphoglucomutase/phosphomannomutase	60
	SPAR46_1575	ridA	endoribonuclease L-PSP family protein	126
	SPAR46_1649	znuA	periplasmic solute binding family protein	71
	SPAR46_1673	ROK	ROK family protein	294
	SPAR46_1692	nanH	sialidase	300
	SPAR46_1715	gntR	bacterial regulatory subunit, GntR family protein	121
	SPAR46_2023	yhaM	OB-fold nucleic acid binding domain protein	308
	SPAR46_2024	rmuC	rmuC family protein	418
	SPAR46_2025	thiN	thiamine pyrophosphokinase	220
	SPAR46_2242	argF	ornithine carbamoyltransferase	338
	SPAR46_2244	yfcC	C4-dicarboxylate anaerobic carrier family protein	503
	SPAR46_1754	yqeH	ribosome biogenesis GTPase	368
	SPAR46_2303	raiA	ribosomal subunit interface protein	182
Genes needing improved description (14.9%)	SPAR46_1934	isl3	hypothetical protein	113
	SPAR46_1746	sir2	hypothetical protein	48
	SPAR46_1615	fer4	hypothetical protein	38
	SPAR46_0749	yutD	hypothetical protein	176
	SPAR46_0899	ycgQ	hypothetical protein	271
	SPAR46_0292	tra8	hypothetical protein	74
	SPAR46_0370	liaF	hypothetical protein	232
	SPAR46_1126	ybaB	hypothetical protein	93
	SPAR46_0423	yloU	hypothetical protein	129
	SPAR46_0432	yloU	hypothetical protein	121
Hypothetical genes (14.9%)	SPAR46_0133	HP	hypothetical protein - DUF1447	77
	SPAR46_0655	HP	hypothetical protein - DUF3042	56
	SPAR46_1193	HP	hypothetical protein - DUF3884	54
	SPAR46_1790	HP	hypothetical protein - DUF4649	73
	SPAR46_1981	HP	hypothetical protein - DUF4231	152
	SPAR46_0494	HP	hypothetical protein - no domain information	118
	SPAR46_0497	HP	hypothetical protein - no domain information	95
	SPAR46_1159	HP	hypothetical protein - no domain information	392
	SPAR46_1297	HP	hypothetical protein - no domain information	105
	SPAR46_1346	HP	hypothetical protein - no domain information	45
